# Postural control and knee joint importance during quiet standing in individuals with anterior cruciate ligament reconstruction

**DOI:** 10.1371/journal.pone.0353774

**Published:** 2026-07-20

**Authors:** Leila Ghazaleh, Nader Farahpour, Peyman Aghaie Ataabadi, Seyyed Morteza Kazemi, Bahram Saleh-Sedghpour, Paul Allard

**Affiliations:** 1 Department of Exercise Physiology, Faculty of Sport Sciences, Alzahra University, Tehran, Iran; 2 Department of Sport Biomechanics, Bu-Ali Sina University, Hamedan, Iran; 3 Department of Sports Injury and Biomechanics, Faculty of Sport Sciences and Health, University of Tehran, Tehran, Iran; 4 Bone, Joint and Related Tissues Research Center, Shaid Beheshti University of Medical Sciences, Tehran, Iran; 5 Department of Educational Sciences, Faculty of Humanities, Shahid Rajaie Teacher Training University, Tehran, Iran; 6 School of Kinesiology, Faculty of Medicine, University of Montreal, Montreal, Canada; Università degli Studi di Milano: Universita degli Studi di Milano, ITALY

## Abstract

There is obscurity about the role of the knee joint during quiet standing (QS) postural control. Examining the knee joint’s contribution during QS in individuals with damaged knee proprioception can help resolve the ambiguity. We investigated whether anterior cruciate ligament reconstruction (ACLR) changes the relative contribution of lower-limb joint motions and postural control during QS. This study included 12 individuals with ACLR and 12 able-bodied individuals. A motion capture system and two force plates were used. The acceleration of the joints, the acceleration of the body center of mass (COM), and the COP parameters were calculated during QS under eyes open (EO) and eyes closed (EC) conditions. In the control group under the EO condition, ankle, knee, and hip accelerations, and under the EC condition, ankle and hip accelerations were primary predictors of COM acceleration. In the ACLR group, under the EO condition, the knee and hip accelerations, and in the EC condition, the ankle, knee, and hip accelerations were prominent predictors. The ACLR group exhibited higher COP parameters than the control group in the closed-eyes condition. The knee plays a vital role in QS postural control. Improving knee proprioception modalities and lower-limb joint coordination is important in ACLR rehabilitation.

## Introduction

The anterior cruciate ligament reconstruction (ACLR) is a common treatment to restore knee stability and function following injury. However, ACLR is associated with impaired postural control and balance due to damage to the related proprioception [1] and impaired static stability [2–4], leading to an increased risk of further injuries.

One key aspect of postural control is the motion of the lower-limb joints, which provide valuable insight into neuromechanical function [5]. A complex interaction between the kinematics of the lower-limb joints seems to govern the whole-body postural control. To our knowledge, the specific contribution of knee motion to the overall pattern of lower-limb movement during QS balance has not been clearly defined. A few authors have investigated the interaction between the knee and the ankle and hip. Jeon et al (2013) concluded that the restriction of the ankle joint threatens postural control more than restrictions on the knee joint when standing [6]. Furthermore, Tsai et al (2020) showed that when the knee motion is restricted, the neuromuscular system relies on an ankle strategy for balance maintenance [7]. However, these studies examined the system’s reaction to an external restriction of a healthy knee. In the case of ACLR, the knee moves freely while its somatosensory feedback and proprioceptive functioning are likely diminished [1]. Therefore, quantifying the motion of the knee and its relationship to other joints and overall sway in this population is a crucial first step.

Authors have postulated that a somatosensory disorder originating from the ankle may increase reliance on input from the visual and vestibular systems [8,9]. The conflict between the inputs from a joint’s somatosensory system (i.e., of the ankle) and the visual/vestibular systems requires CNS processing and integration that may form a compensatory pattern of motion at other joints (i.e., at the hip) [10]. It is plausible that a similar sensory deficit at the knee following ACLR could lead to observable alterations in joint motion patterns, but this adaptation is not well quantified. Understanding these kinematic relationships in individuals with ACLR can be useful in clarifying the importance of the knee joint, clinical assessment, and guiding treatment planning.

Previous studies have identified that the acceleration of lower limb joints is correlated with the accelerations of the COM during quiet standing [11, [12]. Yamamoto et al (2015) suggested that to uncover the physiologically plausible strategy implemented by the CNS for controlling quiet standing, investigations using multi-joint models, including the knee joint, are necessary. They also noted that the knee motion significantly affects COM acceleration [13]. However, the specific nature of these relationships in a population with suspected sensory deficits, like ACLR, remains unknown. The present study aims to test the hypothesis that the statistical correlations between lower-limb joint accelerations and COM acceleration are altered in individuals with ACLR compared to healthy controls during quiet standing postural control.

## Materials and methods

This study recruited 12 male individuals with a history of right-sided ACLR (ACLR group) and 12 male able-bodied individuals without a history of ACLR (control group). All participants were male, had a right-dominant lower limb, and were between 20 and 35 years old. Lower limb dominance was defined as the preferred limb to kick a soccer ball [14]. Participants’ characteristics are presented in Table 1 as mean ± standard deviation. The ACLR participants were recruited from the Bone, Joint, and Related Tissues Research Center of Shahid Beheshti University of Medical Sciences. All participants were recruited between May 1, 2023, and January 10, 2024.

**Table 1:** Demographic characteristics of the subjects (Mean±SD).

Group	n	Height (cm)	Mass (kg)	BMI (kg/m²)
ACLR	12	174.54 ± 7.13	78.51 ± 12.8	25.71 ± 3.36
Control	12	174.12 ± 5.73	75.76 ± 8.92	24.98 ± 2.6

Inclusion criteria for the ACLR group were: a history of right ACLR with hamstring autograft within the last 12–16 months; completion of a post-operative rehabilitation program; and no other orthopedic or neurological disorders. Exclusion criteria for all participants included: any history of surgery or serious injury to either lower limb joints in the six months before the experimentation, current use of medication affecting balance, and any unknown deficits in the visual or vestibular system. Control group participants had no history of ACL injury or surgery. All participants self-reported being physically active.

The study was conducted in accordance with the Helsinki Declaration and approved by the code of ethics IR.SSRI.REC.1400.1239 in the Research Ethics Committee, Institute for Sport Science,

Ministry of Science, Research, and Technology. All informed consent documents were provided in Persian and explained verbally to ensure participants’ full understanding before participation. There were no deviations from the approved study protocol. The study was conducted in Iran, and local collaborators affiliated with Iranian institutions were involved in all stages of the research, including study design, data collection, and data analysis.

### Data collection

An eight-camera motion capture system (Oqus 5 + , Qualisys, Sweden; 250 Hz) and two force plates (9286BA and 9260AA3 Kistler, Switzerland) were used to collect kinematic and kinetic data during the standing balance tests. Forty-three reflective markers (14-mm diameter) were placed over anatomical landmarks of the forearms, upper arms, trunk, pelvis, thighs, shanks, and feet to define a 12-segment model.

Participants were instructed to stand as motionless as possible on the two force plates (separated by 5 cm) with their arms at their sides. The quiet standing balance test was assessed under two conditions: eyes open and eyes closed. Each condition consisted of three 50-second trials with one minute rest between trials. The first 5 seconds of each trial were discarded to eliminate the transient effects from initial stabilization.

### Data processing

Raw kinematic data were filtered using a low-pass Butterworth filter with a cutoff frequency of 5 Hz. Using Visual 3D software (C-Motion, Inc. USA), a 12-segment model was created to estimate the position of the whole-body center of mass (COM). Angular accelerations (⍺) of the dominant (right) limb’s ankle, knee, and hip joints in the sagittal plane were calculated through double differentiation of the joint angle data. To quantify the combined motion of the joints, the time-series angular acceleration data for the right lower limb joints were used to calculate four metrics representing the summed acceleration of different possible combinations of joints [13]:


$$\begin{array}{c} AK=\sum_{i=1}^{n}\left(\alpha_{\text{ankle(i)}}+\alpha_{kne\text{e(i)}}\right) \end{array}$$

$$\begin{array}{c} AH=\sum_{i=1}^{n}(\alpha_{\text{ankle(i)}}+\alpha_{\text{hip(i)}}) \end{array}$$

$$\begin{array}{c} KH=\sum_{i=1}^{n}\left(\alpha_{\text{knee(i)}}+\alpha_{\text{hip(i)}}\right) \end{array}$$

$$\begin{array}{c} \text{AKH}=\sum_{i=1}^{n}\left(\alpha_{\text{ankle(i)}}+\alpha_{\text{knee(i)}}+\alpha_{\text{hip(i)}}\right) \end{array}$$

where n is the sample number of quiet standing duration, ⍺ is angular acceleration, and A, K, and H represent the ankle, knee, and hip joints.

The primary outcome measure for these combined acceleration metrics was their Root Mean Square (RMS) value over each trial, representing the magnitude of the combined signal. The COP was calculated for each foot separately [15]. Postural sway was quantified using the following parameters derived from the anteroposterior (AP) COP trajectory: total excursions (TOTEX_ap_), mean velocity (MVELO_ap_), and the 95% confidence ellipse area (AREA_CE_) [16].

A symmetry index was calculated to assess weight-bearing contribution: SI = (RMS COP_right) / (RMS COP_right + RMS COP_left). The symmetry index provides a measure of the contribution of each limb to postural control [17].

### Statistical analysis

A multivariate analysis of variance (MANOVA) was conducted for each testing condition to determine the effect of group on the dependent variables: the RMS of acceleration for the lower limb joints and the center of pressure (COP). Then, a repeated measures ANOVA (GLM) with two factors was used to analyze within-group factors. The factors included the “eyes factor” with two levels (Eyes-open & eyes-closed) and the “joints factor” with three levels (hip & knee & ankle). An enter regression technique was also utilized to predict the RMS of COM acceleration from the RMS of acceleration of lower limb joints. For regression analysis, each trial was treated as an independent observation to increase the number of data points and improve model estimation in the context of limited sample size [18]. To compare of COP symmetry index between two groups and eye conditions a split plot design was used. The accepted significance level was p < 0.05 for all analyses. Statistical analysis was performed using SPSS 25 package.

## Results

### Lower-limb joint acceleration magnitudes

**Fig 1:**
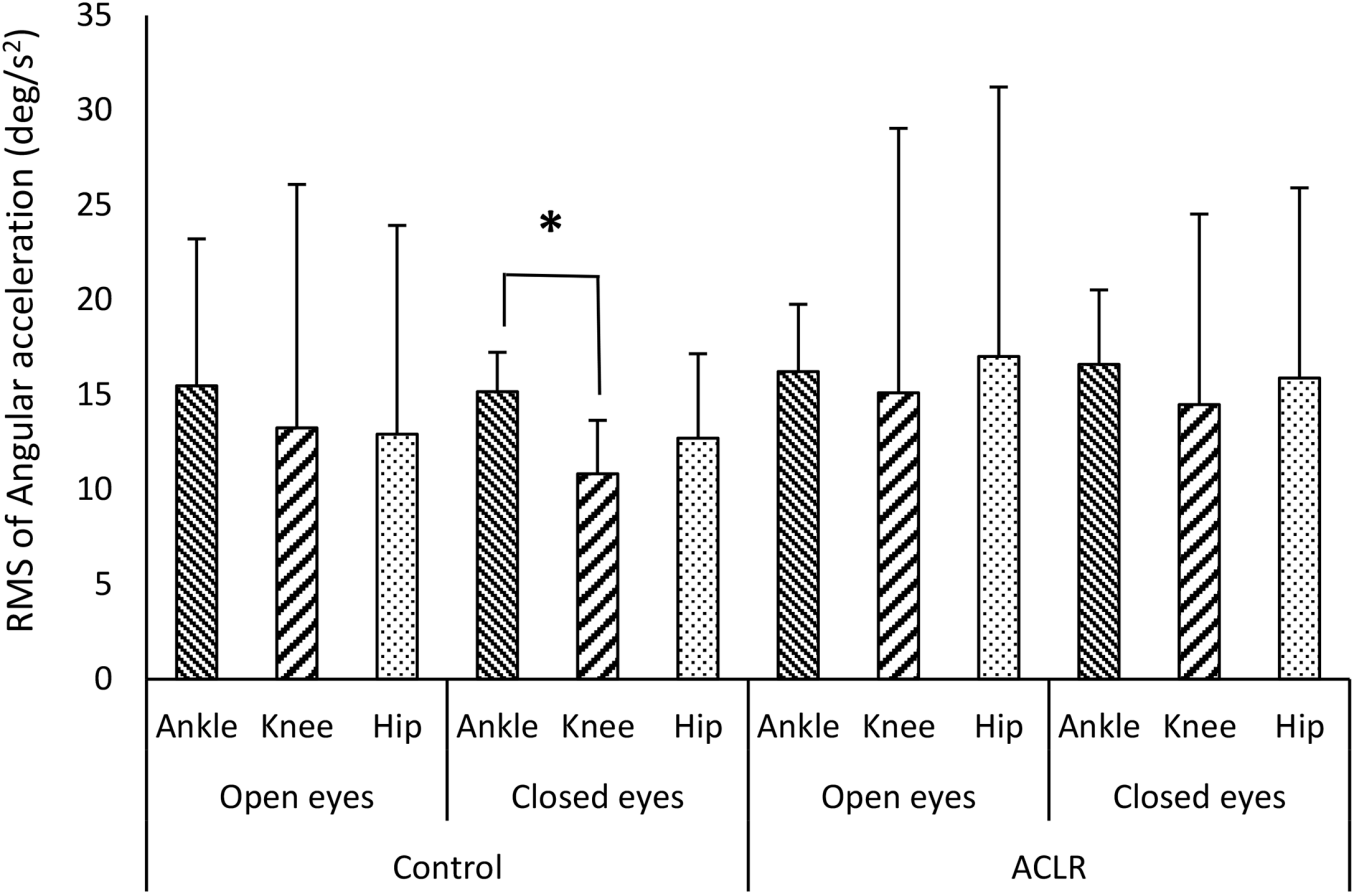
RMS of joints’ acceleration of the ankle, knee, and hip joints (RMS⍺_ankle,_ RMS⍺_knee_, and RMS⍺_hip_) during quiet standing in open-eyes and closed-eye conditions for the control and ACLR groups. *: p=0.000.

The magnitudes of the RMS of angular acceleration for the ankle, knee, and hip joints in both groups are presented in Fig 1. In the control group, no significant differences were found between the RMS of ankle, knee, and hip accelerations during the eyes-open condition (p > 0.05). However, in the eyes closed condition, the RMS⍺_knee_ was significantly lower by 29% than that of the RMS⍺_ankle_ (p = 0.000). In contrast, the ACLR group showed no significant difference in the RMS of the ankle, knee, and hip accelerations under either visual conditions (p > 0.05).

There was no significant main effect of group on the RMS of the ankle, knee, and hip joints’ acceleration in either the eyes-open or eyes-closed conditions.

### Prediction of COM acceleration from joint accelerations

**Control group**: The results of the regression analysis for the control group are detailed in Table 2. In the eyes-open condition, the three-joint model (RMS_(_⍺_ankle +_ ⍺_knee +_ ⍺_hip)_) was the strongest predictor of COM acceleration (Adjusted R^2^ = 0.56; F = 46.09; p = 0.00). Under the eyes-closed condition, all four variables were significant predictors (p < 0.01). The two-joint model RMS_(_⍺_ankle +_ ⍺_hip)_ was the strongest predictor (Adjusted R^2^ = 0.49; F = 34.32; p = 0.000), followed by the three-joint model RMS_(_⍺_ankle+_⍺_knee +_⍺_hip)_ (Adjusted R^2^ = 0.43; F = 27.88; p = 0.000).

**ACLR group:** The results for the ACLR group are presented in Table 3. A different pattern emerged. In the eyes-open condition, the two-joint knee-hip model (RMS_(_⍺_knee+_⍺_hip)_ was the strongest predictor of COM acceleration (Adjusted R² = 0.49, F = 34.44, p = 0.000), followed by the three-joint model RMS_(_⍺_ankle+_⍺_knee+_⍺_hip)_. Under the eyes-closed condition, the three-joint model became the strongest predictor, although the overall predictive power was substantially lower than in the eyes-open condition (Adjusted R^2^ = 0.27; F = 14.11; p = 0.00).

**Table 2:** The regression analysis and model summary in control group in eyes open and closed eyes conditions.

	Predictors	R	R^2^	Adjusted R^2^	F	B	t	Sig
Open-eyes	RMS(⍺_ankle_ + ⍺_knee_)	0.057	0.003	−0.026	0.11	2.34E-05	0.331	0.743
	RMS(⍺_knee_ + ⍺_hip_)	0.203	0.041	0.013	1.455	0	1.206	0.236
	RMS(⍺_ankle_ + ⍺_hip_)	0.120	0.014	−0.015	0.494	4.27E-05	0.703	0.487
	RMS(⍺_ankle_ + ⍺_knee_ + ⍺_hip_)	0.759	0.575	**0.563**	46.088	0.002	6.789	**0.000**
Closed-eyes	RMS(⍺_ankle_ + ⍺_knee_)	0.416	0.173	0.148	7.099	0.001	2.664	0.012
	RMS(⍺_knee_ + ⍺_hip_)	0.629	0.396	0.378	22.253	0.001	4.717	0.000
	RMS(⍺_ankle_ + ⍺_hip_)	0.709	0.502	**0.488**	34.325	0.001	5.859	**0.000**
	RMS(⍺_ankle_ + ⍺_knee_ + ⍺_hip_)	0.671	0.451	0.434	27.877	0.001	5.28	0.00

Predictors (RMS values of algebraic sum of angular acceleration of the joints)

Open-eyes condition: RMS of COM acceleration = -0.010+ (0.002⨯ RMS_(_⍺_ankle+_⍺_knee +_⍺_hip)_)

Closed-eyes condition: RMS of COM acceleration = -0.001+ (0.001⨯ RMS_(_⍺_ankle+_⍺_hip)_)

**Table 3:** The regression analysis and model summary in ACLR group in open and closed eyes conditions.

	Predictors	R	R^2^	Adjusted R^2^	F	B	t	Sig
Open-eyes	RMS(⍺_ankle+_⍺_knee_)	0.022	0.000	-0.029	0.017	1.027E-5	0.130	0.897
	RMS(⍺_knee +_⍺_hip_)	0.709	0.503	**0.489**	34.436	0.001	5.868	**0.000**
	RMS(⍺_ankle+_⍺_hip_)	0.267	0.071	0.044	2.611	0.000	1.616	0.115
	RMS(⍺_ankle+_⍺_knee +_⍺_hip_)	0.703	0.495	**0.480**	33.309	0.001	5.771	**0.000**
Closed-eyes	RMS(⍺_ankle+_⍺_knee_)	0.030	0.001	-0.028	0.030	1.729E-5	0.174	0.863
	RMS(⍺_knee +_⍺_hip_)	0.478	0.228	**0.206**	10.062	0.000	3.172	0.**003**
	RMS(⍺_ankle+_⍺_hip_)	0.229	0.053	0.025	1.885	0.000	1.373	0.179
	RMS(⍺_ankle+_⍺_knee+_⍺_hip_)	0.542	0.293	**0.273**	14.113	0.001	3.757	**0.001**

Predictors (RMS values of algebraic sum of angular acceleration of the joints)

Open-eyes condition: RMS of COM acceleration = 0.005+ (0.001⨯ RMS_(_⍺_knee+_⍺_hip)_)

Closed-eyes condition: RMS of COM acceleration = 0.003+ (0.001 ⨯ RMS_(_⍺_ankle+_⍺_knee+_⍺_hip)_)

### Center of pressure (COP) parameters

The postural sway parameters are summarized in Table 4. A significant main effect was found for both group (p = 0.000) and visual condition (p = 0.000) on COP-based postural sway parameters (Table 4). Post-hoc analyses revealed that the ACLR group exhibited significantly larger postural sway than the control group, with the total excursion (TOTEX) and mean velocity (MVELO) of the COP being 10% and 8.5% higher, respectively (p < 0.03). Furthermore, for both groups, all COP parameters (TOTEX, MVELO, and 95% ellipse area) were significantly higher in the eyes-closed condition compared to the eyes-open condition (p < 0.01).

**Table 4:** Postural sway parameters for right leg in open and closed eyes conditions in control and ACLR groups.

	ACLR		Control	
	Open-eyes	Closed-eyes	Open-eyes	Closed-eyes
TOTEX (mm)	248.31 ± 55.74	315.15 ± 76.94	274.29 ± 49.89	345.21 ± 75.8
MVLO (mm/s)	16.43 ± 3.72	20.85 ± 5.12	17.86 ± 3.23	22.61 ± 5.11
Area-CE (mm^2^)	35.82 ± 16.37	48.09 ± 25.65	41.34 ± 23.41	49.23 ± 25.35

(P < 0.05)

Moreover, the results revealed no significant difference in COP symmetry index between the two groups under both eyes-open (t = 1.31; p = 0.194) and eyes-closed (t = 1.27; p = 209) conditions.

## Discussion

This study aimed to determine if the pattern of lower-limb joint acceleration contributions to COM acceleration and postural control in individuals with ACLR is similar to that in control individuals during quiet standing.

In the control group, while there was no difference in the magnitude (RMS) of individual joint angular acceleration in the open-eyes condition, there was a decrease (of 29%) in knee angular acceleration relative to the ankle during the closed-eyes condition. This suggests a change in strategy where knee motion is minimized when visual input is removed. As noted by Ashtiani et al (2021), the absence of vision can lead to adjustments in postural control, characterized by increased joint rigidity due to the co-contraction of muscle groups, a phenomenon known as postural stiffening [19]. In the present study, the results regarding COM acceleration further support this finding. In the open-eyes condition for the control group, the combined accelerations of all three lower-limb joints were a significant predictor of COM acceleration (Adjusted R^2^ = 0.56). However, in the eyes-closed condition, the able-bodied participants shifted to a strategy where the combined acceleration of the ankle and hip (RMS_(_⍺_ankle+_⍺_hip)_) was the primary predictor. Yamamoto et al (2015) demonstrated that in the eyes-open condition, a three-link inverted pendulum model incorporating knee acceleration overestimated the COM acceleration compared to a two-link inverted pendulum during QS standing balance [13]. In the closed-eyes condition, it is conceivable that in able-bodied participants, the CNS adjusts for increased knee joint stiffness by adopting a postural control strategy where the acceleration of the ankle and hip play dominant roles in governing COM motion. Moreover, the removal of visual input alters the postural control strategies employed by individuals [20]. This CNS attempt to adjust to utilization of joints accelerations did not achieve the same level of postural stability as observed in the eyes-open condition. This is supported by the increased values in postural sway parameters for the control group during the eyes-closed condition, which is consistent with findings from previous studies. When sensory information is limited, sensory reweighting could be insufficient to prevent an increase in postural sway [21].

In the ACLR group, there was no significant difference in the magnitude of the joints’ acceleration between the eyes-closed and eyes-open conditions. Additionally, in the eyes-open condition, the combined accelerations of the knee and hip (RMS_(_⍺_knee +_⍺_hip)_), and in the eyes-closed condition, the combined acceleration of all three joints (RMS_(_⍺_ankle+_⍺_knee+_⍺_hip)_), emerged as the most accurate predictors of the COM acceleration. Individuals with ACLR, often exhibit reduced knee somatosensory feedback and proprioceptive function [1], which may lead to an altered knee acceleration pattern. Piontek et al (2012) suggested that increased erroneous mobility of an unstable knee can introduce erroneous information to the CNS, contributing to misperception of posture and movements in the affected joint [22]. In the eyes-open condition, the CNS may work to regulate the movements of the unstable knee to mitigate postural instability, leading to a predictive model where knee and hip accelerations are most closely associated with COM control. Several studies have demonstrated a significant association between the angular accelerations of lower limb joints and COM control during quiet standing [11, 12]. Additionally, Sassagawa et al (2009) found a significant association between hip motion and COM acceleration during quiet standing in the healthy population [12]. The simultaneous contribution of the hip and knee acceleration to postural control may be facilitated by biarticular thigh muscles, whose role has been reported [23,24]. In the eyes-closed condition, the ACLR group exhibited a shift in strategy, incorporating ankle acceleration alongside knee and hip accelerations; however, the precision of predicting COM acceleration decreased. Okuda et al (2005) underlined the importance of vision in compensating for the diminished contribution of the injured ACL to postural control [25]. In the eyes-closed condition, the ACLR group demonstrated a broader inclusion of joint accelerations to maintain stability. Riemann (2002) postulated that in the absence of joint mechanical stability, compensatory mechanisms could provide the necessary supplemental stability. These compensatory mechanisms can involve motor adaptations at both proximal and distal segments [26]. Despite these attempts to modify the acceleration strategy, the changes were not entirely effective. The findings of the present study indicate that the removal of vision led to a greater decrease in the accuracy of predicting COM acceleration in ACLR patients compared to able-bodied participants.

The COP parameters in the present study supported the kinematic observations. In both the eyes-open and eyes-closed conditions, the ACLR group displayed significantly increased values in COP TOTEX_ap_ and MVELO_ap_ compared to the control group. Dauty et al (2010) reported that the distances covered by COP displacements were greater in ACLR populations compared to healthy controls [27]. Similarly, Kubisz et al (2011) found significantly higher COP sway values in ACLR individuals during QS with eyes closed [3]. The findings suggest that the CNS attempts to regulate posture by adjusting the acceleration patterns of the lower limb joints in ACLR individuals. However, despite these efforts, postural control in ACLR individuals during QS is not as effective as in able-bodied individuals.

The results of the COP symmetry index in the present study indicated that individuals with ACLR, similar to the healthy control group, engage both lower limbs equally in postural control. This finding emphasizes that the reason for the different joint acceleration pattern between individuals with ACLR and healthy individuals is not due to reduced weight bearing on the operated lower limb during quiet standing.

A limitation of the present study is the lack of information about muscle activation and joint moments. Kinetic variable measurements would be beneficial for neuromechanical control of the systematic movement and joint control patterns. Furthermore, the calculated metrics for combined joint acceleration represent their magnitude but do not quantify the temporal relationships between joints. Future research should employ techniques such as vector coding to directly quantify inter-joint coordination in this population. Another limitation is the relatively small sample size for regression analyses, as well as the treatment of each trial as an independent observation, which may violate the assumption of independence and should be considered when interpreting the predictive models.

## Conclusion

The acceleration of the knee joint contributes to the control of COM acceleration during QS in eyes-open conditions. In the absence of visual input, the relative contribution of knee acceleration is reduced, and the ankle and hip play a more dominant role. In individuals with ACLR, the pattern of joint acceleration contributions is altered. The combined accelerations of the knee and hip are prominent predictors of COM control when vision is available, while the accelerations of all three joints are utilized when vision is removed, albeit less effectively. The altered joint acceleration patterns observed in individuals with ACLR, along with their reduced ability to predict COM motion, highlight the vital role of the knee joint in QS postural control and the presence of persistent sensorimotor deficits. Therefore, rehabilitation should focus on enhancing knee proprioception and lower-limb joint coordination in individuals with ACLR.
